# Heart failure in Somalia: a scoping review of the epidemiology, etiology, clinical characteristics, management, outcomes, and evidence gaps

**DOI:** 10.3389/fcvm.2026.1960696

**Published:** 2026-09-28

**Authors:** Abdihamid Hirsi Abdulle, Khalid Hussein Ulusow, Abdullahi Abdi Yusuf, Abdisalam Ismail Hassan, Said Abdi Mohamed, Salma Mohamed Ahmed, Iqra Abdirazak Kabayare, Deka Ahmed Abdi, Aden Mohamed Hirsi, Ayan Mohamed Hassan

**Affiliations:** 1 Faculty of Medicine and Surgery, Somali National University, Mogadishu, Somalia; 2 Benaadir Research, Consultancy and Evaluation Center, Mogadishu, Somalia; 3 Research and Education Unit, Mogadishu Virtual Academy (MOVA), Mogadishu, Somalia; 4 Department of Neurology, Mogadishu Somali–Türkiye Recep Tayyip Erdoğan Training and Research Hospital, Mogadishu, Somalia

**Keywords:** cardiovascular disease, echocardiography, epidemiology, health systems, heart failure, scoping review, Somalia

## Abstract

**Background:**

Heart failure is an important cause of morbidity, hospitalisation, disability, and mortality, but evidence from Somalia remains fragmented and largely confined to individual hospital-based studies. This scoping review mapped the available evidence on the epidemiology, aetiologies, clinical and diagnostic characteristics, management, outcomes, and research gaps relating to heart failure in Somalia.

**Methods:**

PubMed/MEDLINE, Scopus, and Embase were searched from inception to 27 July 2026. Backward citation searching was conducted on 3 August 2026. Original studies reporting heart-failure-specific findings from Somalia, including Somaliland and Puntland, were eligible. Screening was conducted independently by two reviewers. Data were charted using a structured form and verified by a second reviewer. Findings were synthesised descriptively without meta-analysis because of substantial heterogeneity and possible participant overlap.

**Results:**

The searches identified 111 records, of which 56 were duplicates. Fifty-five records underwent title and abstract screening, 13 reports were assessed in full text, and 12 studies published between 2011 and 2025 were included. Eleven studies were associated with a single tertiary hospital in Mogadishu, and no study provided nationally representative or multicentre evidence. Reported aetiologies and associated conditions included hypertension, ischaemic heart disease, valvular disease, cardiomyopathy, anaemia, thyroid dysfunction, renal disease, and thiamine-deficiency wet beriberi. Echocardiography was the principal diagnostic method, but heart-failure definitions and ejection-fraction thresholds varied substantially. Management data were limited mainly to selected populations with thyroid dysfunction, left ventricular thrombus, or wet beriberi. Outcome reporting was sparse and included in-hospital mortality, first-year outcomes in a thrombus cohort, length of stay, stroke, thrombus resolution, and exploratory prognostic indices.

**Conclusions:**

The evidence on heart failure in Somalia is emerging but remains geographically concentrated, methodologically heterogeneous, and insufficient to estimate national burden, treatment coverage, or long-term outcomes. Prospective multicentre studies and a staged heart-failure registry using standardised diagnostic definitions, clear denominators, treatment indicators, and longitudinal follow-up are needed.

## Introduction

Heart failure is a major global clinical syndrome and public-health problem, with substantial effects on hospitalization, symptom burden, disability, healthcare utilization, and premature mortality (1). Contemporary global estimates indicate that tens of millions of people live with heart failure worldwide, including about 56.5 million prevalent cases in 2021, and the burden is expected to remain important as populations age and underlying cardiovascular risk profiles change (1, 2). Heart failure is also heterogeneous in both pathophysiology and clinical expression, arising from multiple underlying cardiac disorders and presenting across phenotypes defined by ventricular function, remodeling, and congestion rather than as a single disease entity (3). This heterogeneity has practical importance because the distribution of etiologies, clinical characteristics, and outcomes varies markedly across regions and health systems, limiting direct extrapolation from evidence generated predominantly in high-income settings (3, 4).

In Sub-Saharan Africa, heart failure appears to differ from the pattern typically described in high-income countries in age at presentation, etiological profile, and short-term outcomes (5). African cohorts and reviews consistently indicate a younger affected population, often by roughly two decades, alongside high case fatality and frequent rehospitalization (5, 6). The etiologic spectrum remains broad: hypertensive heart disease, dilated and other cardiomyopathies, and rheumatic or other valvular heart disease continue to contribute prominently, while ischemic heart disease is increasingly reported within an ongoing epidemiological transition rather than replacing older causes entirely (7). Coexisting renal dysfunction, anemia, diabetes, and other non-cardiac comorbidities are also common in African heart-failure populations (8). Available regional evidence further suggests that infectious, endocrine, and nutritional contributors may remain relevant in selected settings, while diagnostic classification is often complicated by limited access to comprehensive imaging and laboratory evaluation (9). Together, these features argue for country-specific synthesis rather than assuming that findings from other regions, or even from Sub-Saharan Africa as a whole, adequately capture local heart-failure patterns (6).

Within Somalia, including Somaliland and Puntland where relevant, the published heart-failure literature is emerging but remains fragmented across institution-based studies and clinically selected populations. Somali studies have addressed general hospital heart-failure cohorts, phenotypes defined by ejection fraction, and selected comorbid or mechanistic questions such as anemia, thyroid dysfunction, and left ventricular thrombus in reduced-ejection-fraction heart failure (10). Related Somali cardiovascular studies have also examined acute myocardial infarction complicated by heart failure, hypertensive emergencies with acute heart failure, echocardiographic patterns of cardiovascular disease, angiographic coronary disease among patients with systolic dysfunction, and cardiovascular complications among patients receiving maintenance hemodialysis (11, 12). Historical and contextual evidence additionally supports the relevance of nutritional cardiac syndromes such as wet beriberi as potentially reversible causes of heart-failure presentations in resource-constrained settings (12). However, the available Somali evidence appears largely hospital-based, geographically concentrated, and methodologically heterogeneous, with differing denominators, case definitions, diagnostic approaches, and outcome measures; it should therefore not be interpreted as nationally representative of heart failure in Somalia (9).

Despite this growing body of work, the Somali literature has not yet been systematically mapped across epidemiology, etiologies, clinical and diagnostic characteristics, management, outcomes, methodological limitations, and research gaps. This is an important gap because the available evidence spans heterogeneous observational designs, selected hospital cohorts, mixed cardiovascular populations, varying heart-failure definitions, and diverse clinical endpoints, making a conventional effect-focused review less suitable than an evidence-mapping approach. To our knowledge, no previous review has comprehensively mapped the full spectrum of heart-failure evidence generated in Somalia. This scoping review aimed to map and synthesize the available evidence on the epidemiology, etiologies, clinical and diagnostic characteristics, management, outcomes, and evidence gaps relating to heart failure in Somalia.

## Methods

### Review design and reporting

This study was conducted as a scoping review to map the nature, extent, and characteristics of the available evidence on heart failure in Somalia. A scoping-review design was considered appropriate because the available literature was expected to include heterogeneous study populations, designs, clinical settings, heart-failure definitions, diagnostic approaches, and reported outcomes. The review was conducted in accordance with the Joanna Briggs Institute methodology for scoping reviews and reported according to the Preferred Reporting Items for Systematic Reviews and Meta-Analyses Extension for Scoping Reviews (PRISMA-ScR) (13, 14). Eligibility criteria were structured using the Population–Concept–Context framework recommended for scoping reviews. A completed PRISMA-ScR checklist is provided as a Supplementary file.

### Protocol and registration

The review was not prospectively registered, and no protocol was published or made publicly available. The review question, eligibility criteria, information sources, and study-selection procedures were defined before formal screening commenced. Any refinements made during screening or data charting were intended to clarify the application of the predefined criteria rather than alter the scope or objective of the review.

### Review question and eligibility criteria

The review addressed the following question**:** What evidence is available on the epidemiology, etiologies, clinical and diagnostic characteristics, management, outcomes, health-system implications, and research gaps relating to heart failure in Somalia?

The eligible population comprised people of any age who received a diagnosis of acute or chronic heart failure within Somalia. Eligible conditions included congestive heart failure; heart failure with reduced, mildly reduced, or preserved ejection fraction; left-sided, right-sided, or biventricular heart failure; and cardiomyopathy accompanied by clinical heart failure. Heart-failure diagnoses could be established through clinical assessment, recognized diagnostic criteria, diagnostic coding, echocardiography, or another clearly reported clinical method. The definitions and ejection-fraction classifications applied by the authors of the original studies were retained rather than retrospectively reclassifying participants according to contemporary thresholds. Studies involving mixed clinical populations were included only when findings concerning participants with heart failure were reported separately or could be reliably extracted. No publication-date restriction was applied. The databases were searched from their dates of inception to 27 July 2026. No language filter was applied during the database searches. Reports were required to provide sufficient information to determine eligibility and extract heart-failure-specific findings.

The concept of interest encompassed evidence relating to the occurrence and distribution of heart failure; etiologies, associated conditions, and cardiovascular risk factors; clinical presentation; laboratory, electrocardiographic, echocardiographic, and angiographic characteristics; diagnostic practices; heart-failure phenotypes; pharmacological and non-pharmacological management; hospitalization; mortality; complications; length of stay; readmission; follow-up; prognostic indicators; health-service limitations; and research gaps.

The geographical context comprised studies in which the relevant clinical event, heart-failure presentation, diagnosis, or initial healthcare encounter occurred within Somalia, including Somaliland and Puntland. Eligibility was determined by the geographical setting of the clinical evidence rather than by participant nationality. Studies involving subsequent referral outside Somalia remained eligible only when the referred participants originated from the same Somalia-based clinical episode and the reported heart-failure findings could be clearly linked to that episode. Studies of Somali diaspora populations receiving care entirely outside Somalia were excluded unless findings concerning participants assessed or treated within Somalia were separately available.

Eligible sources included original quantitative, qualitative, and mixed-methods research, including cross-sectional studies, cohort studies, case-control studies, clinical trials, registries, surveillance studies, outbreak investigations, and case series containing at least five eligible participants. Individual case reports and case series with fewer than five eligible participants were excluded. Reviews, meta-analyses, editorials, commentaries, perspectives, protocols, and letters without original data were also excluded. Additional exclusions were studies conducted outside Somalia, studies in which heart failure was mentioned only incidentally, and mixed-population studies from which heart-failure-specific findings could not be extracted**.**

### Information sources

PubMed/MEDLINE, Scopus, and Embase were searched on 27 July 2026. The reference lists of all included articles were subsequently examined through backward citation searching on 3 August 2026 to identify potentially eligible studies that had not been retrieved through the database searches. No updated database search was conducted after 27 July 2026. No additional grey-literature database or general web-search platform was searched.

### Search strategy

The database searches combined controlled vocabulary, where available, with free-text terms representing the two principal concepts of heart failure and the Somali geographical context. Heart-failure terms included variations of heart failure, cardiac failure, congestive heart failure, and relevant abbreviations. Geographic terms included Somalia, Somali, Somaliland, and Puntland. Boolean operators, field tags, truncation, and database-specific indexing terms were adapted to the requirements of each database.

The search strategies were designed to maximise sensitivity because terminology used to describe heart failure and the Somali geographical context could vary across studies. No study-design, publication-date, or language filter was applied. The complete reproducible search strategies for PubMed/MEDLINE, Scopus, and Embase, including the search platform, exact search date, field tags, indexing terms, free-text terms, Boolean operators, truncation, and number of records retrieved, are provided in Additional file 1. The reported strategies reproduce the searches as originally conducted and were not retrospectively modified after study selection.

### Selection of sources of evidence

All retrieved references were imported into Rayyan, a web-based platform for organizing and screening records in evidence syntheses (15). Duplicate records were identified and resolved before title and abstract screening. Records remaining after deduplication were screened against the predefined eligibility criteria, and potentially relevant reports were obtained for full-text assessment. Full texts were evaluated against the Population–Concept–Context criteria and the specified source-type requirements. The principal reason for exclusion was documented for every report excluded at the full-text stage. Title and abstract screening was conducted independently by two reviewers (A.H.A and K.H.U). The same reviewers independently assessed the full texts of potentially eligible reports against the predefined Population–Concept–Context criteria and source-type requirements. Disagreements at either screening stage were resolved through discussion and consensus. Where consensus could not be reached, a third reviewer (A.A.Y) was available for adjudication.

Following full-text selection, the reference lists of all included articles were screened for additional potentially eligible evidence. Reports identified through backward citation searching were subjected to the same eligibility criteria as records identified through the electronic database searches. The complete selection process was documented using a PRISMA flow diagram (16).

### Data charting process

A structured data-charting form was developed in Microsoft Excel and aligned with the review question, eligibility criteria, and review domains. The form captured study characteristics, participant and clinical information, heart-failure definitions, diagnostic findings, management, outcomes, methodological limitations, reporting inconsistencies, and potential cohort overlap. Data were charted from the full-text articles by one reviewer (A.H.A) and independently checked for completeness and accuracy against the original articles by a second reviewer (K.H.U). Discrepancies were resolved through discussion and consensus.

Data were charted at the study level. For studies involving mixed clinical populations, the overall parent-cohort sample was recorded separately from the heart-failure-specific denominator. Only findings explicitly reported for participants with heart failure, or findings that could be reliably derived from reported numerators and denominators, were charted as heart-failure evidence. The terminology, diagnostic criteria, and phenotype definitions used by the original authors were retained.

When numerical values differed across an article's abstract, narrative text, tables, or figures, the inconsistency was documented rather than silently resolved. Raw numerators and denominators were prioritised where available. Values calculated by the reviewers from reported data were labelled as review-derived. Missing information was recorded as “not reported” and was not inferred from related articles or general clinical knowledge.

Potential participant overlap was assessed by comparing institution, location, recruitment period, study population, eligibility criteria, and author group. Studies originating from the same institution during overlapping periods were retained when they addressed distinct eligible review questions, but their samples were not assumed to represent independent or unique patients.

### Data items

The charted study characteristics included author, publication year, study objective, study design, recruitment period, city or region, hospital or healthcare setting, sampling method, inclusion criteria, parent population, overall sample size, and heart-failure-specific denominator.

Participant and clinical variables included age, sex, heart-failure definition, diagnostic method, ejection fraction, heart-failure phenotype, suspected etiology, cardiovascular risk factors, comorbidities, presenting symptoms and signs, laboratory findings, electrocardiographic findings, echocardiographic findings, and coronary angiographic findings.

Management variables included reported medications, procedures, anticoagulation, nutritional treatment, and other heart-failure-related interventions. Outcome variables included in-hospital and follow-up mortality, hospitalization duration, prolonged admission, complications, stroke, thrombus resolution, readmission, follow-up duration, and reported prognostic indicators. Methodological variables included sampling limitations, denominator clarity, completeness of diagnostic evaluation, missing data, follow-up completeness, reporting inconsistencies, author-reported limitations, and possible cohort overlap**.**

### Critical appraisal of sources of evidence

A formal methodological quality or risk-of-bias appraisal was not conducted because the purpose of this scoping review was to map the nature, range, and characteristics of the available evidence rather than estimate pooled effects or produce certainty-of-evidence judgements. According to JBI methodological guidance, critical appraisal in a scoping review should be determined by the review objective and is not mandatory in every review (13).

Nevertheless, methodological and reporting characteristics relevant to interpretation were systematically charted. These included study design, sampling approach, clinical setting, heart-failure case definition, clarity of the eligible denominator, completeness of diagnostic assessment, missing-data reporting, duration and completeness of follow-up, internal numerical inconsistencies, and possible participant overlap. These characteristics were summarised descriptively and were not converted into numerical quality scores or overall risk-of-bias ratings**.**

### Synthesis and presentation of results

The extracted evidence was synthesized using descriptive numerical summaries and structured narrative synthesis. Studies were organized according to their design, setting, population, and principal area of contribution. Findings were grouped into the following review domains: study characteristics and scope of the available literature; epidemiology and hospital-based occurrence; heart-failure definitions and phenotypes; etiologies, associated conditions, and risk factors; clinical and diagnostic characteristics; management; outcomes and prognostic indicators; methodological limitations; and evidence gaps.

Direct heart-failure cohorts were distinguished from studies in which heart failure represented a subgroup of a broader clinical population. Evidence from patients selected because of acute myocardial infarction, hypertensive crisis, hemodialysis, left ventricular thrombus, abnormal echocardiographic findings, or referral for coronary angiography was interpreted within its original sampling frame and was not generalized to all patients with heart failure in Somalia.

Meta-analysis was not undertaken because of substantial heterogeneity in study populations, designs, clinical settings, heart-failure definitions, diagnostic methods, denominators, and outcome measures. Possible overlap among cohorts recruited from the same institution during similar periods provided an additional reason not to aggregate sample sizes or pool estimates. The samples reported across the included studies were therefore not summed and presented as a national total of unique patients.

Hospital-based proportions were reported according to the denominators used in the individual studies and were not interpreted as estimates of national or population prevalence. Findings were presented as counts, percentages, and ranges where appropriate, with clear identification of the population and denominator to which each estimate applied. No inferential statistical analysis, pooled effect estimation, assessment of publication bias, or certainty of evidence grading was performed.

The results were presented through a PRISMA flow diagram, one main evidence table, three supplementary tables, and a narrative synthesis.

### Use of generative artificial intelligence

ChatGPT by OpenAI was used only to support language refinement and editorial organisation. It was not used to determine study eligibility, independently extract data, perform statistical analysis, or make final scientific decisions. All factual statements, numerical data, citations, and interpretations were checked by the authors against the original sources, and the authors take full responsibility for the final manuscript**.**

## Results

### Study selection

The database searches identified 111 records, comprising 27 from PubMed, 33 from Scopus, and 51 from Embase. After removal of 56 duplicate records, 55 records underwent title and abstract screening, of which 42 were excluded. Thirteen reports were assessed in full text, and one was excluded because it was an individual case report or a case series containing fewer than five eligible participants. Twelve studies met the eligibility criteria and were included in the review. Backward citation searching did not identify any additional eligible studies. The study-selection process is shown in Figure 1.

**Figure 1:**
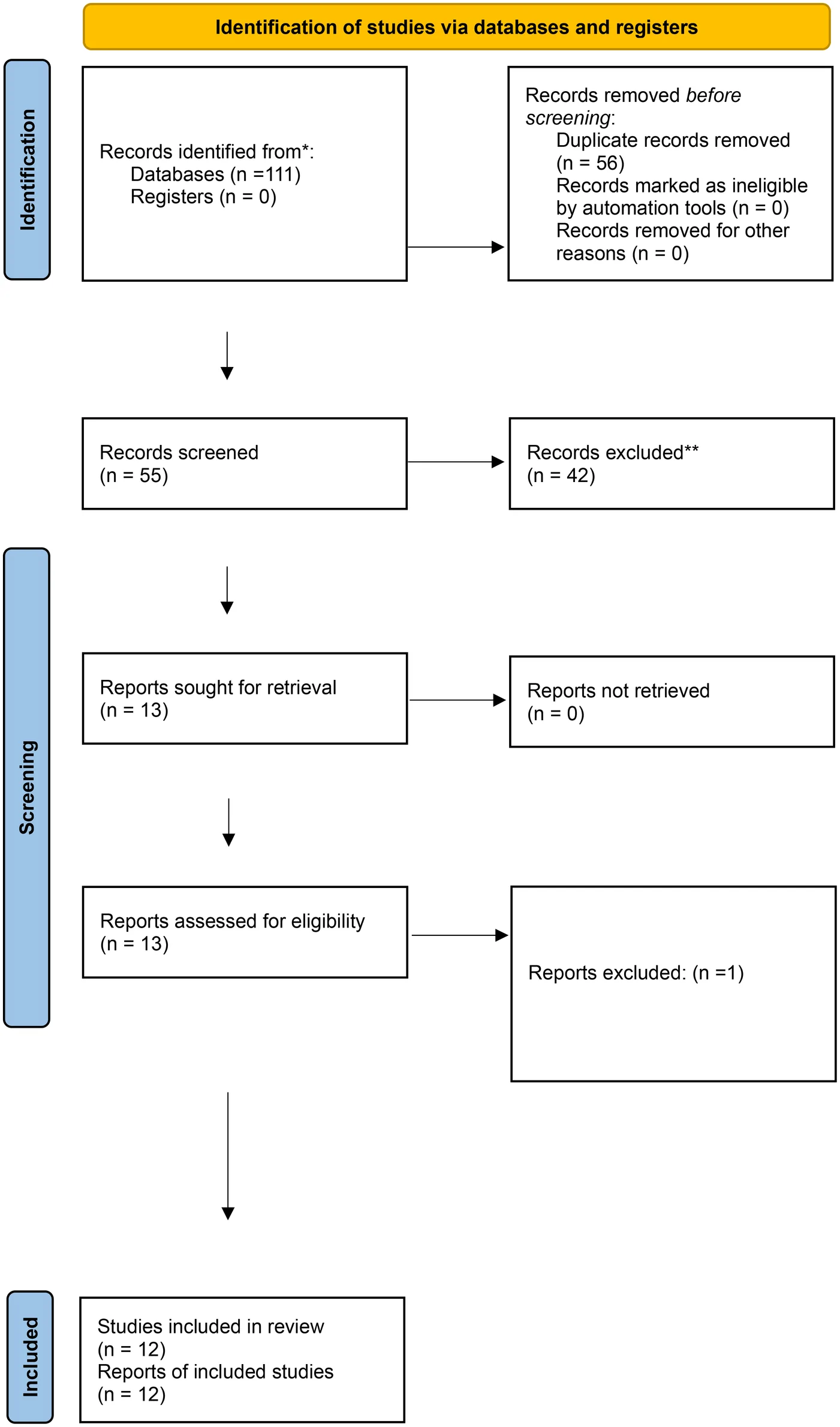
PRISMA 2020 flow diagram showing the identification, screening, eligibility assessment, and inclusion of studies. Source: Page MJ et al., BMJ. 2021;372:n71 (16).

### Characteristics of included studies

The 12 included studies were published between 2011 and 2025, although 11 were published from 2021 onwards. All used quantitative designs. Nine were retrospective studies, including cross-sectional record reviews, observational cohorts, and an echocardiography registry; two used prospective designs; and one was an outbreak investigation incorporating case-control and clinical record-review components. The corresponding study periods extended from April 2009 to April 2025. The studies addressed a broad range of heart-failure-related questions, including general clinical profiles and aetiologies, anaemia, thyroid dysfunction, acute myocardial infarction complicated by heart failure, hypertensive emergencies, cardiovascular disease among patients receiving haemodialysis, left ventricular thrombus in HFrEF, echocardiographic patterns, angiographic coronary artery disease, and the prognostic performance of the HALP and CALLY indices. The evidence was geographically and institutionally concentrated. All studies generated at least part of their eligible data in Mogadishu, and 11 of the 12 reports originated from, or were attributable to, the Mogadishu Somali Turkish Training and Research Hospital and its emergency, cardiology, dialysis, outpatient, or inpatient services. The remaining study investigated a wet-beriberi outbreak among African Union troops stationed in Mogadishu. Initial identification and management occurred at the AMISOM Level II Hospital in Mogadishu, while severely affected patients were subsequently referred to Nairobi for additional assessment. The study was retained because the outbreak and initial clinical episode occurred in Somalia, although its findings concerned foreign military personnel rather than the Somali civilian population. No eligible study provided multicentre national coverage or substantial evidence from healthcare facilities outside Mogadishu. Seven studies investigated direct heart-failure populations, including general HF, acute HF, HFrEF, angiography-selected systolic HF, and HFrEF complicated by left ventricular thrombus. Five studies evaluated broader clinical populations in which heart failure formed a separately extractable subgroup, including acute myocardial infarction, hypertensive crisis, haemodialysis, abnormal echocardiograms, and wet beriberi. HF-specific denominators varied considerably, ranging from six patients with echocardiographic changes suggestive of right-sided HF in the beriberi investigation to 351 patients meeting an author-defined HFrEF criterion in the echocardiography registry. Most cohorts consisted predominantly of middle-aged or older adults, although the beriberi cohort was considerably younger, with a mean age of 29 years among hospitalised participants. Sex distributions varied, but male predominance was reported in most studies. The characteristics of the included studies are presented in Table 1. Because several reports originated from the same institution during overlapping recruitment periods, study sample sizes were not combined as a total number of unique patients.

**Table 1:** Characteristics of studies included in the scoping review.

Study ID	Study	Design	Study period	Setting	Population type	Parent sample/source population	HF-specific sample	Age	Sex	Main focus
S01	Mohamud et al., 2022 - Baseline characteristics, risk factors and etiology of HF	Hospital-based retrospective cross-sectional study	May-Oct 2021	Emergency Unit, Mogadishu Somali Turkish Training and Research Hospital, Mogadishu	Direct HF cohort	4,500 emergency-unit patients screened	155 patients with definite HF	Reported inconsistently: abstract 65.9 ± 14 y; Table 1 reports 62 ± 14 y (range 20–92)	Female 91/155 (58.7%)	Baseline characteristics, risk factors, etiologies, and EF phenotypes
S02	Jeele et al., 2022 - Anemia among patients with HF	Retrospective record-based study	Jan-Mar 2021	Cardiology outpatient and emergency departments, Mogadishu Somali Turkish Training and Research Hospital	Direct HF cohort	200 selected HF records; broader source population not reported	200 patients with HF	Mean 62.26 ± 14 y (range 20–92)	Male 114/200 (57.0%)	Prevalence and patterns of anemia in HF
S03	Mohamud, 2022 - Acute myocardial infarction	Prospective cohort study	Dec 2020-Nov 2021	Emergency Department, Mogadishu Somali Turkish Training and Research Hospital	Mixed AMI cohort; HF reported as a complication/subgroup	23,147 ED attendees; 3,526 with chest pain; 97 first AMI cases	35/97 (36.1%) developed HF	Results: 55.5 ± 17.1 y (range 18–95); abstract reports 54.2 ± 12.1 y	Male 67/97 (69.1%)	HF and EF impairment complicating AMI
S04	Mohamud et al., 2022 - Thyroid dysfunction in HF	Single-center retrospective cohort study	Jan 2019-Jan 2021	Cardiology outpatient and emergency departments, Mogadishu Somali Turkish Training and Research Hospital	Direct HF cohort	250 selected HF records	250 patients with HF	Mean 59.8 ± 14 y	Male 159/250; paper reports 63.2% although 159/250 = 63.6%	Thyroid dysfunction prevalence, types, and associated HF characteristics
S05	Watson et al., 2011 - Beriberi outbreak among AU troops	Outbreak investigation with case-control and clinical sub-study	26 Apr 2009-1 May 2010	AMISOM Level II Hospital, Base Camp, Mogadishu; severe cases referred to Aga Khan University Hospital, Nairobi	Mixed wet-beriberi outbreak with separately reported right-sided HF subgroup	241 outbreak cases; 33 hospitalized in Nairobi; 31 underwent echocardiography	6/31 (19.4%) had echocardiographic changes suggestive of right-sided HF	Hospitalized subgroup mean 29 y (range 24–39)	Hospitalized subgroup 33/33 male	Thiamine-deficiency wet beriberi, right-sided HF, outbreak response
S06	Mohamud, 2023 - Hypertensive crisis	Prospective cross-sectional study	Nov 2020-Apr 2021	Emergency Clinic, Mogadishu Somali Turkish Training and Research Hospital	Mixed hypertensive-crisis cohort; acute HF as end-organ damage subgroup	6,239 emergency patients screened; 128 hypertensive crises; 70 hypertensive emergencies	32/70 (45.7%) hypertensive emergencies had acute HF	Mean 56.5 ± 16.9 y (range 24–98)	Male 76/128 (59.4%)	Acute HF as a complication of hypertensive emergency
S07	Mohamud et al., 2023 - Cardiovascular events in hemodialysis	Single-center retrospective cross-sectional study	May-Oct 2021	Dialysis center, Mogadishu Somali Turkish Training and Research Hospital	Mixed ESRD/hemodialysis cohort with HF subgroup	224 ESRD/HD records; 200 included; 59 had at least one CVD	Approximately 16/59 (27.1% of CVD cases; about 8.0% of all 200) had HF	Mean 54 ± 17.5 y (range 18–88)	Male 106/200 (53.0%)	HF among patients receiving routine hemodialysis
S08	Abdi et al., 2022 - Left ventricular thrombus in HFrEF	Retrospective cross-sectional study	Jan-Dec 2021	Cardiology Department, Mogadishu Somali Turkish Training and Research Hospital	Direct HFrEF plus LVT cohort	810 echocardiograms; study flow is internally inconsistent	40 adults with LVEF <40% and echocardiographically confirmed LVT	Mean 51 ± 15 y	Male 30/40 (75.0%)	LVT characteristics, etiology, anticoagulation, and outcomes
S09	Alici and Genc, 2021 - Echocardiographic spectrum of CVD	Retrospective descriptive observational registry	1 Jan 2019-1 Jan 2020	Outpatient cardiology clinic, tertiary training hospital, Mogadishu	Mixed abnormal-echocardiogram cohort with HFrEF subgroup	6,782 screened; 1,140 with abnormal echocardiograms included	351/1,140 (30.8%) met the authors’ HFrEF definition	Median 60 y (IQR 42–70)	Male 646/1,140 (56.7%)	Echocardiographic spectrum and author-defined HFrEF
S10	Abdi et al., 2025 - Angiographic CAD in systolic HF	Retrospective cross-sectional record-based study	Jan 2023-Jan 2025	Mogadishu Somali Turkiye Training and Research Hospital	Direct HFrEF/HFmrEF cohort selected for coronary angiography	Broader source population not reported	169 HF patients undergoing coronary angiography	Mean 54.3 ± 10.9 y	Male 56/169 (33.1%)	Angiographic prevalence, severity, and predictors of CAD
S11	Aydin and Abdi, 2025 - HALP score in acute HF	Retrospective observational study	Jan 2024-Apr 2025	Mogadishu Somali Turkiye Recep Tayyip Erdogan Training and Research Hospital	Direct hospitalized acute-HF cohort	Broader source population not reported	222 hospitalized acute-HF patients	Mean/median not reported; age >=65 y: 42/222 (19.1%)	Male 138/222 (62.7%)	HALP score, prolonged hospitalization, and in-hospital mortality
S12	Arin et al., 2025 - CALLY score and mortality in HF	Retrospective observational cohort study	Jan 2022-Dec 2024	Mogadishu Somali Turkish Training and Research Hospital (identified by affiliations/ethics; methods retain a [Hospital Name] placeholder)	Direct hospitalized HF cohort	Broader source population not reported	220 hospitalized HF patients	Survivors 58.09 ± 6.18 y; non-survivors 68.35 ± 8.16 y	Male: 46.0% of survivors and 42.0% of non-survivors	CALLY score, HF phenotype, length of stay, and in-hospital mortality

### Scope and distribution of the available evidence

The included evidence was heterogeneous in its populations, clinical focus, and contribution to the review objectives. Seven studies directly enrolled patients with heart failure and examined general clinical profiles, anaemia, thyroid dysfunction, left ventricular thrombus in HFrEF, angiographic coronary artery disease in systolic HF, and nutritional–inflammatory prognostic indices among hospitalised HF populations (10, 17–22).

Five studies examined broader clinical populations from which HF-specific findings were separately extractable. These comprised cohorts of patients with acute myocardial infarction, hypertensive crisis, end-stage renal disease receiving haemodialysis, abnormal echocardiographic findings, and wet beriberi (12, 23–25).

Overall, the literature was concentrated on hospital-based clinical characterisation, ejection-fraction phenotypes, aetiologies and associated conditions, echocardiographic and angiographic findings, selected complications, and prognostic markers. Management and outcome information was reported in a smaller subset of studies, notably those examining thyroid dysfunction, left ventricular thrombus, wet beriberi, prolonged hospitalisation, and inflammatory or nutritional prognostic indices (10, 17–19, 24). No included study primarily evaluated population-level incidence, community burden, quality of life, readmission, continuity of care, device therapy, multidisciplinary HF services, or national HF service capacity. Consequently, individual studies contributed to different review domains, and findings from selected clinical cohorts were interpreted within their original sampling frames rather than as representative estimates for the wider Somali population.

### Heart-failure definitions and phenotypes

Heart-failure ascertainment varied substantially across the included studies. One general HF cohort used the Framingham criteria, defining definite HF as at least two major criteria or one major plus two minor criteria, supported by cardiologist assessment and echocardiography. In that cohort, HFpEF accounted for 74/155 patients (47.7%), HFrEF for 54/155 (34.9%), and HFmrEF for 27/155 (17.4%). A second study identified HF from hospital records and ICD-10 coding and classified patients using echocardiographic ejection fraction; HFpEF was reported in 95/200 patients (47.5%), HFrEF in 68/200 (34.0%), and mid-range EF in 37/200 (18.5%). Another record-based HF cohort classified 147/250 patients (58.8%) as having HFrEF and 103/250 (41.2%) as having HFpEF, although the study did not clearly describe the original clinical diagnostic criteria and contained inconsistent wording for its ejection-fraction categories (17, 20, 21).

Definitions were less detailed in studies where HF occurred within broader clinical populations. In the hypertensive-crisis study, acute cardiac failure was treated as evidence of acute target-organ damage and was reported in 32/70 patients with hypertensive emergencies; acute pulmonary oedema was reported separately in 23/70 and was not combined with the HF group. Among patients receiving haemodialysis, HF was defined by abnormal left-ventricular filling or an age-inappropriate mitral E:A pattern and/or systolic dysfunction with an ejection fraction below 50%, but no phenotype-specific results were provided (12, 23, 25). Several studies focused on selected HF presentations. The wet-beriberi investigation did not apply formal HF criteria but identified echocardiographic changes suggestive of right-sided HF in 6/31 examined patients, while pulmonary hypertension was reported separately in 4/31. The left-ventricular-thrombus study included only patients with clinical HF, echocardiographically confirmed thrombus, and LVEF below 40%; all 40 participants therefore had HFrEF, with a mean LVEF of 25.25% ± 6.97%. In the echocardiography registry, the authors defined HFrEF using symptoms, signs, cardiovascular risk factors, or abnormal electrocardiographic findings together with LVEF below 50%. Under this author-defined category, 351/1,140 patients with abnormal echocardiograms were classified as having HFrEF, including 59 with LVEF of 41%–49% and 292 with LVEF of 40% or lower. This definition therefore encompassed ranges corresponding to both HFrEF and HFmrEF under contemporary classifications. The angiography study separately defined HFrEF as LVEF below 40% and HFmrEF as 41%–49%, but reported the two phenotypes as a combined systolic-HF cohort without providing phenotype-specific counts (10, 22, 24, 26).

The two prognostic studies used guideline-based HF identification but differed in phenotype reporting. The HALP study enrolled adults hospitalised with acute HF reportedly confirmed according to European Society of Cardiology guidance but did not report the distribution of HFrEF, HFmrEF, and HFpEF. The CALLY study required symptoms or signs, elevated pro–B-type natriuretic peptide, and objective echocardiographic dysfunction; among 220 patients, 38.2% had HFrEF, 25.5% had HFmrEF, and 36.4% had HFpEF (18, 19). Study-specific HF definitions and phenotypes are summarized in Supplementary Table 1.

### Etiologies, risk factors, and associated conditions

The reported causes and associated conditions of HF varied across study populations and clinical settings. In a general hospital-based HF cohort, hypertensive heart disease was the most frequently reported aetiology, affecting 70/155 patients (45.2%), followed by ischaemic heart disease in 40/155 (25.8%), valvular heart disease in 20/155 (12.9%), cardiomyopathy in 12/155 (7.7%), arrhythmia in 9/155 (5.8%), and congenital heart disease in 4/155 (2.6%). Hypertension was also the most common recorded risk factor in this cohort, affecting 102/155 patients (65.8%), followed by ischaemic heart disease in 47/155 (30.3%), diabetes mellitus in 41/155 (26.4%), hyperlipidaemia in 36/155 (23.2%), and thyroid disease in 25/155 (16.1%). A separate HF cohort similarly reported hypertension in 99/200 patients (49.5%), ischaemic heart disease in 56/200 (28.0%), valvular heart disease in 34/200 (17.0%), and cardiomyopathy in 11/200 (5.5%) (20, 21).

Systemic and metabolic conditions were examined in several direct HF cohorts. Anaemia was identified in 59/200 patients with HF (29.5%), most commonly iron-deficiency anaemia, which affected 37/200 (18.5%). Vitamin B12 deficiency and anaemia of chronic disease were each reported in 9/200 patients (4.5%), while folate deficiency and mixed nutritional deficiencies were less frequent. Thyroid dysfunction was reported in 89/250 HF patients (35.6%), including subclinical hypothyroidism, overt hypothyroidism, low-triiodothyronine syndrome, overt hyperthyroidism, and subclinical hyperthyroidism. Within the thyroid-dysfunction subgroup, hypertension, ischaemic heart disease, valvular disease, and cardiomyopathy were also commonly recorded (17). Ischaemic and non-ischaemic myocardial disease were prominent in selected systolic-HF populations. Among 40 patients with HFrEF and echocardiographically confirmed left ventricular thrombus, 23/40 (57.5%) had ischaemic cardiomyopathy and 17/40 (42.5%) had non-ischaemic or dilated cardiomyopathy. Diabetes was present in 21/40 patients (52.5%), hypertension in 18/40 (45.0%), atrial fibrillation in 12/40 (30.0%), chronic kidney disease in 7/40 (17.5%), and hypothyroidism in 5/40 (12.5%). In a separate cohort of 169 patients with HFrEF or HFmrEF selected for coronary angiography because coronary artery disease was suspected, angiographic CAD was detected in 90/169 (53.3%), including severe disease in 39/169 (23.1%). These findings apply to highly selected HFrEF, thrombus, and angiography populations and should not be interpreted as estimates for the wider Somali HF population (10, 22).

Several broader clinical cohorts demonstrated HF in association with specific underlying conditions. HF developed in 35/97 patients admitted with acute myocardial infarction (36.1%), including 27/73 patients with ST-elevation myocardial infarction and 8/24 with non-ST-elevation myocardial infarction. Acute cardiac failure was also reported in 32/70 patients with hypertensive emergencies (45.7%). Among patients receiving maintenance haemodialysis, HF represented 27.1% of the 59 patients with recorded cardiovascular disease, corresponding to approximately 16 cases; this percentage should not be applied to the full cohort of 200 dialysis patients. In the wet-beriberi outbreak investigation, thiamine deficiency associated with a restricted and monotonous diet was linked to congestive and right-sided cardiac manifestations, with echocardiographic changes suggestive of right-sided HF in 6/31 examined patients (12, 23–25). In one hospitalised acute-HF cohort, diabetes was recorded in 123/222 patients, hypertension in 118/222, and chronic kidney disease in 29/222. These variables were reported as comorbid conditions within a prognostic study rather than as independently established causes of HF (19). Overall, the available studies described a mixture of hypertensive, ischaemic, valvular, cardiomyopathic, renal, endocrine, haematological, and nutritional conditions, but the relative national contribution of these factors could not be determined from the predominantly hospital-based and selected cohorts.

### Clinical and diagnostic characteristics

Clinical and diagnostic reporting varied across the included studies, and several reports focused on selected laboratory or imaging findings rather than providing a complete clinical description of HF. In the general HF cohort, the median LVEF was 45% (range 30%–50%), and the median pulmonary arterial pressure was 25 mmHg (range 20–40 mmHg). Echocardiography supported HF classification, whereas B-type natriuretic peptide testing was not used. In the wet-beriberi investigation, prominent manifestations included oedema, dyspnoea, palpitations, and signs of cardiorespiratory compromise. Among the 31 hospitalised patients who underwent echocardiography, 6/31 had findings suggestive of right-sided HF and 4/31 had pulmonary hypertension; cardiac catheterisation was performed in 10 patients (21, 24).

Laboratory evaluation was reported most comprehensively in studies examining anaemia, thyroid dysfunction, and nutritional–inflammatory indices. Anaemia was identified in 59/200 patients with HF (29.5%), with iron-deficiency anaemia accounting for 37/200 (18.5%). Vitamin B12 deficiency and anaemia of chronic disease were each reported in 9/200 patients (4.5%), while folate deficiency and combined nutritional deficiencies were less frequent. In the thyroid study, serum thyroid-stimulating hormone, free triiodothyronine, and free thyroxine were assessed, and thyroid dysfunction was identified in 89/250 patients (35.6%). The acute-HF prognostic studies incorporated pro–B-type natriuretic peptide, C-reactive protein, albumin, lymphocyte count, haemoglobin, and platelet measurements; elevated pro–B-type natriuretic peptide was reported in 216/222 participants in the HALP cohort (17–20).

Echocardiography was the principal imaging method used to classify HF phenotypes and characterize structural abnormalities. In the selected cohort of 40 patients with HFrEF and left ventricular thrombus, the mean LVEF was 25.25% ± 6.97%, and 24/40 patients (60.0%) had an LVEF of 25% or lower. Thrombi were located at the left ventricular apex in 21/40 patients, the inferior wall in 11/40, and the anterior wall in 8/40; a left ventricular aneurysm was reported in 15/40. In the echocardiography registry, 351/1,140 patients with abnormal echocardiograms met the authors’ HF definition based on LVEF below 50%, comprising 59 patients with LVEF of 41%–49% and 292 with LVEF of 40% or lower. However, age, symptoms, and structural findings were not separately reported for this HF subgroup (10, 26).

Coronary angiographic evidence was available from one selected cohort of 169 patients with HFrEF or HFmrEF referred for angiography because coronary artery disease was suspected. Any angiographic CAD was identified in 90/169 patients (53.3%), including minor disease in 35/169, moderate disease in 16/169, and severe disease in 39/169 (23.1%). Normal coronary arteries were reported in 79/169 patients. Among those with angiographically detected CAD, single-vessel disease was reported in 51 patients, double-vessel disease in 24, triple-vessel disease in 13, and left-main involvement in 2. The mean LVEF was reported as 28.6% ± 6.0%, although the article contained inconsistencies between the narrative and tabulated ejection-fraction values. These angiographic findings apply only to patients selected for invasive investigation and do not represent the frequency of CAD among all Somali patients with HF (22). Etiologies, associated conditions, and major clinical and diagnostic findings are presented in Supplementary Table 1.

### Management patterns

Management information was limited and inconsistently reported across the included studies. Most reports focused on clinical characteristics, associated conditions, diagnostic findings, or prognostic markers rather than providing a comprehensive description of HF treatment. Consequently, only three studies contributed directly extractable treatment data for eligible HF populations or HF-related cardiac presentations.

Among 89 patients with both HF and thyroid dysfunction, ACE inhibitors were reported in 97.8%, beta-blockers in 98.9%, spironolactone in 77.5%, and furosemide in 80.7%. Thyroid-directed treatment included unspecified thyroid medications in 19.1% and levothyroxine in 38.2%. These percentages applied only to the thyroid-dysfunction subgroup and should not be interpreted as treatment rates among all 250 patients in the parent HF cohort (17).

In the wet-beriberi outbreak, symptomatic soldiers were treated with intramuscular thiamine after thiamine deficiency was suspected. This approach was subsequently adopted for clinically affected cases. Mass supplementation was introduced for all troops using oral thiamine at 10 mg daily for two weeks, followed by 1 mg daily for four weeks. Dietary measures included increasing the proportion of vegetables and meat in the troops’ meals. These interventions formed part of an outbreak-control response rather than routine management of a general Somali HF population (24).

In the selected cohort of 40 patients with HFrEF and left ventricular thrombus, 37 received long-term oral anticoagulation. Warfarin was used in 19/40 patients (47.5%), rivaroxaban in 8/40 (20.0%), and dabigatran in 10/40 (25.0%). Two patients died before oral anticoagulation could be initiated, while one patient did not receive anticoagulation because of a bleeding diathesis (10).

Treatment data reported in the acute myocardial infarction and hypertensive-crisis studies related to their entire parent cohorts and were not separately available for participants with HF. Similarly, HF-specific treatment was not reported in the haemodialysis, echocardiography-registry, angiography, HALP, or CALLY studies. Treatment history in the angiography cohort was described as inconsistently documented, while the two prognostic studies did not present medication-use results. These studies were therefore not used to estimate HF-specific medication patterns (12, 18, 19, 22, 23, 25, 26). Reported management and clinical outcomes are summarized in Supplementary Table 2.

### Outcomes and prognostic indicators

Outcome reporting was limited to four studies and varied substantially in population, follow-up period, and outcome definition. In the wet-beriberi outbreak, 4/33 hospitalised patients (12.1%) died within 48 h, while the remaining 29 recovered and were discharged. No further deaths were reported after mass thiamine supplementation was introduced. These outcomes concerned the hospitalised outbreak subgroup of African Union troops and were not reported specifically for the six patients with echocardiographic evidence of right-sided HF. In the HFrEF cohort with left ventricular thrombus, 12/40 patients (30.0%) died during the first year. Stroke occurred in 8/40 patients (20.0%), comprising six ischaemic and two haemorrhagic strokes, while peripheral arterial disease was reported in 6/40 (15.0%) (10, 24). Repeat echocardiography was available for 28 of the 40 patients with left ventricular thrombus. Thrombus resolution occurred in 17/28 reassessed patients (60.7%), while thrombus persisted in 11/28 (39.3%); four of the persistent thrombi decreased in size. These proportions use the repeat-echocardiography denominator rather than the full study population because 12 participants did not undergo reassessment (10). In the acute-HF HALP study, 86/222 patients (38.7%) had a prolonged hospital stay of more than seven days, and 21/222 (9.5%) died during hospitalisation. A HALP score above the study threshold was associated with prolonged admission in multivariable analysis (adjusted OR 10.19, 95% CI 2.49–41.63; *p* = 0.002). Previous stroke was also associated with prolonged hospitalisation (OR 8.44, 95% CI 1.15–61.88; *p* = 0.035). However, the HALP score demonstrated poor standalone discrimination for prolonged hospital stay, with an AUC of 0.511. Published mortality percentages for the short- and long-stay groups were internally inconsistent; therefore, only the overall mortality count was retained (19). In the CALLY study, 26/220 hospitalised patients with HF (11.8%) died in hospital. Non-survivors had a longer mean hospital stay than survivors (4.96 ± 1.84 versus 3.05 ± 1.06 days) and a lower mean CALLY score (1.27 ± 0.72 versus 1.71 ± 0.60; *p* < 0.001). Increasing age was associated with mortality in the multivariable model (HR 1.105, 95% CI 1.031–1.202; *p* = 0.004). The source article reported an inverse estimate between CALLY and in-hospital mortality (HR 0.495, 95% CI 0.281–1.010; *p* = 0.047). However, because the confidence interval included 1.0 while the reported *p* value was below 0.05, the statistical outputs were internally inconsistent and the association was considered inconclusive (18). Although 30-day readmission was listed as a secondary outcome, corresponding results were not reported (18). Overall, the available outcome evidence was limited to in-hospital mortality, first-year outcomes in a selected thrombus cohort, hospital-stay duration, thromboembolic complications, and two nutritional–inflammatory indices. No included study reported population-level survival, routine post-discharge mortality, consistently measured readmission, quality of life, functional recovery, or long-term outcomes across an unselected Somali HF cohort. Reported management, clinical outcomes, and prognostic indicators are summarized in Supplementary Table 2.

### Methodological characteristics and evidence gaps

the evidence base was methodologically limited and highly concentrated. Most studies were retrospective, single-centre, and hospital-based, and 11 of the 12 reports originated from the same tertiary hospital in Mogadishu. Several cohorts were recruited during overlapping periods and involved similar author groups and clinical services, creating a possibility of participant overlap. Study populations were also frequently selected, including patients with acute myocardial infarction, hypertensive emergencies, haemodialysis, left ventricular thrombus, abnormal echocardiograms, or referral for coronary angiography. Heart-failure definitions, ejection-fraction thresholds, denominators, diagnostic work-up, and follow-up periods varied across studies, while some reports contained internally inconsistent numerical values. Important evidence gaps remained. No included study provided a population-based estimate of HF incidence or prevalence, multicentre national surveillance, or substantial evidence from regions outside Mogadishu. Data were also limited on guideline-recommended treatment, medication access and adherence, readmission, quality of life, functional status, post-discharge care, long-term survival, device therapy, and multidisciplinary HF services. The concentration of evidence in selected hospital populations therefore limited national generalisability and prevented quantitative pooling. Study-level methodological limitations, reporting inconsistencies, and possible cohort overlap are detailed in Supplementary Table 3.

## Discussion

### Principal findings

This scoping review identified 12 studies describing heart failure in Somalia, but the available evidence was predominantly hospital-based, single-centre, and concentrated in Mogadishu. The studies were heterogeneous in design, population, diagnostic approach, heart-failure definition, ejection-fraction threshold, and outcome reporting. Several reports also originated from the same institution during overlapping periods, meaning that participant overlap could not be excluded and study samples could not be combined as a unique national population. Consequently, the existing evidence does not permit reliable estimation of the incidence or prevalence of heart failure in Somalia. Across the included studies, hypertension, ischaemic heart disease, valvular disease, cardiomyopathy, anaemia, thyroid dysfunction, renal disease, and nutritional deficiency were reported as relevant aetiologies or associated conditions. Echocardiography was the principal diagnostic tool, although definitions of HFrEF, HFmrEF, and HFpEF were inconsistent across studies. Evidence on treatment was limited and mainly restricted to selected populations, including patients with thyroid dysfunction, left ventricular thrombus, and wet beriberi. Outcome reporting was similarly sparse, with only a few studies addressing mortality, length of stay, thrombus resolution, stroke, or prognostic indices. Overall, the review demonstrates that Somalia has an emerging but fragmented heart-failure evidence base, with substantial gaps in population-level burden estimates, standardised diagnosis, treatment reporting, multicentre surveillance, and long-term follow-up.

### Comparison with sub-Saharan African evidence

The Somali hospital-based evidence broadly fits the wider sub-Saharan African literature in showing that heart failure in the region often presents at a younger age than in high-income settings, but it does not justify any claim about a uniquely Somali age profile or national epidemiologic pattern. Multicountry and African reviews consistently describe heart failure in sub-Saharan Africa as affecting patients roughly a decade earlier than in high-income countries, with substantial regional heterogeneity in causes, comorbidity burden, and prognosis (3, 27, 28).

Contemporary African data also reinforce that this is not a static pattern: younger presentations coexist with an epidemiologic transition toward older, more cardiometabolic phenotypes in some settings (29, 30).

The etiologic mix reported from Somali studies is likewise compatible with, but not equivalent to, broader African experience. Across major African registries and reviews, hypertensive heart disease, dilated cardiomyopathy, and rheumatic or other valvular disease remain prominent contributors to heart failure, although their relative importance varies markedly by country, referral pathway, and diagnostic depth (9). THESUS-HF identified rheumatic heart disease as the third most common cause of acute heart failure overall, while younger Nigerian patients more often showed dilated cardiomyopathy and rheumatic disease than older patients (30). East African evidence also supports continuing relevance of rheumatic disease, even as its burden differs across populations and study frames (9). In that context, the Somali signals for valvular disease and cardiomyopathy are plausible, but they should be interpreted as referral-centre observations rather than national rank-order estimates.

Comparison with neighbouring East African evidence further illustrates both shared patterns and the limitations of the Somali evidence base. A recent multicentre Kenyan registry involving 795 patients across three tertiary hospitals reported a predominantly middle-aged heart-failure population, frequent hypertension, a predominance of reduced ejection fraction, and measurable short-term mortality (31), while an Ethiopian systematic review identified substantial acute heart-failure mortality with marked between-study heterogeneity (32). These findings demonstrate that clinically important variation exists even within East Africa and that differences between countries may reflect case mix, referral pathways, diagnostic capacity, and study design as much as underlying epidemiology. In contrast, 11 of the 12 Somali studies originated from a single tertiary institution in Mogadishu, preventing comparable assessment of geographic variation within Somalia. This contrast strengthens the need for multicentre prospective surveillance capable of capturing differences across Mogadishu, Somaliland, Puntland, and other regions.

An important point of comparison is the increasing recognition of ischaemic heart disease in African heart failure cohorts. Earlier African datasets emphasized predominantly non-ischaemic causes, but more recent work shows a rising contribution from ischaemic disease alongside urbanization, hypertension, diabetes, and obesity (28). The Somali review's identification of ischaemic disease within selected cohorts is therefore directionally consistent with continental transition, but ascertainment was limited and partly dependent on highly selected angiographic assessment. For that reason, the Somali data cannot determine whether ischaemic disease is under-recognized, increasing, or preferentially captured in tertiary care.

The Somali studies also align with African literature in showing that heart failure rarely occurs in isolation. Reviews and cohorts across sub-Saharan Africa describe substantial contributions from renal dysfunction, anaemia, diabetes, and other comorbidities to clinical complexity and poor outcomes (9, 29). African reviews further note that context-specific contributors, including endocrine and nutritional conditions, remain relevant where diagnostic pathways are incomplete and competing disease burdens persist (9). The Somali findings on thyroid dysfunction and wet beriberi are therefore clinically important reminders that heart failure evaluation in low-resource settings must remain etiologically broad.

Outcome comparisons should also be cautious. Across sub-Saharan Africa, both short-term and longer-term outcomes remain poor, with high mortality and frequent rehospitalisation after discharge (9). However, the Somali studies were too heterogeneous in population selection, outcome definition, and follow-up duration to support direct comparison with THESUS-HF, INTER-CHF, or national registries. The main inference is not that Somali outcomes are better or worse, but that Somalia currently lacks the standardized follow-up data needed to make that comparison reliably.

### Clinical and diagnostic interpretation

A major interpretive issue is inconsistency in how heart failure was defined and phenotyped. Current guideline frameworks emphasize heart failure as a clinical syndrome supported by objective evidence, while ejection fraction is used to classify phenotype rather than to establish the diagnosis in isolation (33, 34). Variation in clinical criteria, ICD coding, echocardiographic definitions, and EF thresholds therefore reduces comparability across Somali studies and increases the risk of misclassifying HFrEF, HFmrEF, and HFpEF (6).

This problem is especially important where HFrEF was defined as LVEF below 50%, because that approach combines patients who would conventionally be separated into HFrEF and HFmrEF categories under contemporary guideline-based classifications (33). Since treatment evidence, prognosis, and complication profiles differ across EF phenotypes, future Somali studies should adopt standard EF categories and clearly distinguish acute from chronic heart failure presentations.

Echocardiography was appropriately central in the Somali evidence base because it is essential for assessing ventricular function, valvular disease, and complications such as visible thrombus.

Beyond phenotype classification, the available evidence also highlights an uneven diagnostic pathway. Echocardiography was the dominant non-invasive modality, whereas natriuretic-peptide testing was reported only in selected cohorts and was absent from the general heart-failure cohort; coronary angiography was reported in a highly selected population referred because coronary artery disease was suspected (19, 21, 22). These investigations address different clinical questions and should not be interpreted as interchangeable indicators of diagnostic capacity. Future Somali heart-failure studies should therefore report not only whether biomarkers, echocardiography, and invasive investigations were performed, but also their availability, clinical indications, timing, and diagnostic yield. Such reporting would help distinguish true disease patterns from differences produced by referral pathways and access to diagnostic testing.

### Management and outcome gaps

The main management gap in the Somali literature is incomplete treatment reporting rather than demonstrable absence of care. Contemporary ESC and AHA/ACC/HFSA guidance supports foundational HFrEF therapy with renin–angiotensin system inhibition or ARNI, evidence-based beta-blockers, mineralocorticoid receptor antagonists, and SGLT2 inhibitors, with diuretics for congestion and additional therapies guided by phenotype and comorbidity (35, 36). Recent African reviews nevertheless show persistent underdocumentation and incomplete uptake of guideline-directed therapy, including low reporting of SGLT2 inhibitor use and limited post-discharge care (9). The limited treatment evidence also prevents assessment of how closely contemporary guideline-directed medical therapy is implemented in Somalia. Where treatment was reported, ACE inhibitors and beta-blockers were prominent in a selected subgroup with thyroid dysfunction (17), whereas ARNI and SGLT2 inhibitors were not consistently documented across the included literature despite their established role in contemporary HFrEF therapy (35, 36). No included study systematically reported the eligibility, availability, or use of implantable cardioverter-defibrillators, cardiac resynchronisation therapy, or other advanced heart-failure technologies. Importantly, non-reporting should not be interpreted as evidence that these treatments or devices are unavailable; rather, it represents an important evidence gap regarding access to and implementation of advanced heart-failure care. Future multicentre studies should capture medication class, dose and titration, contraindications, device eligibility and use, and barriers to access alongside clinical outcomes.

Within that context, the absence of consistent Somali data on ACE inhibitor, ARB, ARNI, beta-blocker, MRA, SGLT2 inhibitor, iron therapy, ischemic secondary prevention, discharge planning, and follow-up precludes any firm estimate of treatment coverage or quality. This distinction is important because lack of reporting should not be interpreted as lack of prescribing, availability, or access. The selected evidence on anticoagulation for left ventricular thrombus and thiamine replacement for wet beriberi remains clinically relevant, but these are cause-specific observations that cannot be generalized to unselected heart failure populations.

Outcome interpretation is similarly limited. Mortality estimates cannot be pooled because study populations, denominators, follow-up periods, and endpoints differed materially, a problem that also complicates broader heart failure epidemiology when definitions and ascertainment vary across studies (37). African cohorts nevertheless suggest that readmission and death remain frequent after hospitalization and may improve with closer post-discharge optimization and follow-up, underscoring the importance of measuring these outcomes systematically in Somalia (9). HALP and CALLY should therefore be regarded as exploratory only; they require prospective external validation and should not substitute for standardized clinical documentation, guideline-based treatment reporting, and longitudinal follow-up.

### Health-system implications and research priorities

The clearest next step is a prospective multicentre Somali heart failure registry. African registry experience shows the value of standardized, prospective data collection across sites, and also shows how strongly case mix and outcomes depend on geography, referral patterns, and local diagnostic capacity (6). For Somalia, this should include hospitals outside Mogadishu, including Somaliland and Puntland, and should use standardized heart failure and LVEF definitions, clear HF-specific denominators, consistent etiologic classification, and transparent reporting of overlapping recruitment periods.

A staged design beginning in referral hospitals is likely to be more feasible than immediate national surveillance. THESUS-HF II provides a useful model by combining patient-level data with hospital-level information on staffing, diagnostics, and treatment availability, as well as follow-up to discharge, 30 days, and later time points (3). A minimum Somali dataset should include demographics, location and referral pathway, acute versus chronic presentation, phenotype and LVEF, suspected etiology and precipitating factors, comorbidities, laboratory and imaging findings, inpatient treatment, discharge medication, length of stay, and outcomes including mortality, readmission, quality of life, adherence, and functional status. Without this structure, it will remain difficult to distinguish true epidemiologic patterns from artefacts of referral, documentation, and selected testing.

### Strengths and limitations

To our knowledge, this review is the first comprehensive map of published heart failure evidence from Somalia. Its strengths include predefined PCC criteria, searching of three databases with backward citation searching, independent screening by two reviewers, structured extraction and verification, separation of parent-study and HF-specific denominators, preservation of original definitions, explicit identification of inconsistencies and possible cohort overlap, and avoidance of inappropriate pooling. These design choices are particularly important in a sparse and methodologically heterogeneous literature, where over-aggregation would create spurious precision. The review also has important limitations. Only three databases were searched, no formal grey-literature search was undertaken, the search was not updated after 27 July 2026, and one historical report was not retrieved, so non-indexed or difficult-to-access evidence may have been missed. No formal risk-of-bias appraisal was performed. More importantly, the underlying evidence base comprised only 12 studies and was predominantly retrospective, single-centre, and concentrated in Mogadishu, with heterogeneous definitions, selected populations, and possible participant overlap. Accordingly, the review cannot estimate national burden, treatment coverage, or long-term outcomes, and its main contribution is to define the current evidence gap as clearly as the available studies allow. One included outbreak investigation involved foreign military personnel stationed in Mogadishu, and severely affected participants underwent part of their cardiac assessment after referral to Nairobi. Although the clinical episode originated in Somalia, the findings may have limited applicability to the Somali civilian population.

## Conclusions

The available evidence on heart failure in Somalia is growing but remains limited, fragmented, and predominantly derived from hospital-based studies conducted in Mogadishu. Existing studies describe a heterogeneous clinical spectrum involving hypertensive, ischaemic, valvular, cardiomyopathic, renal, endocrine, haematological, and nutritional conditions, but their selected populations, inconsistent diagnostic definitions, variable ejection-fraction thresholds, incomplete treatment reporting, and possible cohort overlap prevent reliable estimation of the national burden of heart failure. Evidence on guideline-based treatment, readmission, quality of life, functional recovery, post-discharge care, and long-term survival is particularly scarce. Future research should prioritise prospective multicentre studies and a staged national heart-failure registry using standardised diagnostic and phenotype definitions, clearly reported denominators, consistent aetiological classification, treatment indicators, and longitudinal outcomes. Expanding evidence generation beyond Mogadishu will be essential for developing a more representative understanding of heart failure and informing context-appropriate cardiovascular care in Somalia.

## Data Availability

The original contributions presented in the study are included in the article/Supplementary Material, further inquiries can be directed to the corresponding author.
