# Supplementary material for: Heart failure in Somalia: a scoping review of the epidemiology, etiology, clinical characteristics, management, outcomes, and evidence gaps
Source: Front Cardiovasc Med. 2026 Sep 28;13:1960696. doi: 10.3389/fcvm.2026.1960696 (PMC13656846; doi:10.3389/fcvm.2026.1960696)
Supplement: Supplementary file 1 [file Supplementaryfile1.docx]

**Additional file 1**

**Complete database search strategies**

*Heart Failure in Somalia: A Scoping Review of the Epidemiology, Etiology, Clinical Characteristics, Management, Outcomes, and Evidence Gaps*

**Search date:** 27 July 2026. **Coverage:** From database inception to the search date. **Limits:** No publication-date, study-design, or language filters were applied.

The following strategies reproduce the searches as originally conducted. Controlled vocabulary was combined with free-text terms for heart failure and the Somali geographical context, including major cities, regions, and affiliation fields.

# Search strategy 1: PubMed/MEDLINE

**Database/platform:** PubMed | **Date searched:** 27 July 2026 | **Records retrieved:** 27

(
 "Heart Failure"[Mesh]
 OR "heart failure"[tiab]
 OR "cardiac failure"[tiab]
 OR "myocardial failure"[tiab]
 OR "cardiac insufficiency"[tiab]
 OR "cardiac decompensation"[tiab]
 OR "congestive heart failure"[tiab]
 OR "congestive cardiac failure"[tiab]
 OR "acute heart failure"[tiab]
 OR "chronic heart failure"[tiab]
 OR "decompensated heart failure"[tiab]
 OR "acute decompensated heart failure"[tiab]
 OR "left ventricular failure"[tiab]
 OR "right ventricular failure"[tiab]
 OR "biventricular failure"[tiab]
 OR "systolic heart failure"[tiab]
 OR "diastolic heart failure"[tiab]
 OR "heart failure with reduced ejection fraction"[tiab]
 OR "heart failure with mildly reduced ejection fraction"[tiab]
 OR "heart failure with preserved ejection fraction"[tiab]
 OR HFrEF[tiab]
 OR HFmrEF[tiab]
 OR HFpEF[tiab]
 OR CHF[tiab]
 OR ADHF[tiab]
)
AND
(
 "Somalia"[Mesh]
 OR Somalia[tiab]
 OR Somaliland[tiab]
 OR Puntland[tiab]
 OR Mogadishu[tiab]
 OR Hargeisa[tiab]
 OR Garowe[tiab]
 OR Garoowe[tiab]
 OR Bosaso[tiab]
 OR Bossaso[tiab]
 OR Burao[tiab]
 OR Burco[tiab]
 OR Borama[tiab]
 OR Boorama[tiab]
 OR Baidoa[tiab]
 OR Baydhabo[tiab]
 OR Kismayo[tiab]
 OR Kismaayo[tiab]
 OR Galkayo[tiab]
 OR Gaalkacyo[tiab]
 OR Beledweyne[tiab]
 OR Beletweyne[tiab]
 OR Banadir[tiab]
 OR Benadir[tiab]
 OR Jubaland[tiab]
 OR Galmudug[tiab]
 OR Hirshabelle[tiab]
 OR "Federal Republic of Somalia"[tiab]
 OR Somalia[ad]
 OR Somaliland[ad]
 OR Puntland[ad]
 OR Mogadishu[ad]
 OR Hargeisa[ad]
 OR Garowe[ad]
 OR Garoowe[ad]
 OR Bosaso[ad]
 OR Bossaso[ad]
 OR Burao[ad]
 OR Burco[ad]
 OR Borama[ad]
 OR Boorama[ad]
 OR Baidoa[ad]
 OR Baydhabo[ad]
 OR Kismayo[ad]
 OR Kismaayo[ad]
 OR Galkayo[ad]
 OR Gaalkacyo[ad]
 OR Beledweyne[ad]
 OR Beletweyne[ad]
 OR Banadir[ad]
 OR Benadir[ad]
 OR Jubaland[ad]
 OR Galmudug[ad]
 OR Hirshabelle[ad]
)

# Search strategy 2: Scopus

**Database/platform:** Scopus | **Date searched:** 27 July 2026 | **Records retrieved:** 33

TITLE-ABS-KEY(
 "heart failure"
 OR "cardiac failure"
 OR "myocardial failure"
 OR "cardiac insufficiency"
 OR "cardiac decompensation"
 OR "congestive heart failure"
 OR "congestive cardiac failure"
 OR "acute heart failure"
 OR "chronic heart failure"
 OR "decompensated heart failure"
 OR "acute decompensated heart failure"
 OR "left ventricular failure"
 OR "right ventricular failure"
 OR "biventricular failure"
 OR "systolic heart failure"
 OR "diastolic heart failure"
 OR "heart failure with reduced ejection fraction"
 OR "heart failure with mildly reduced ejection fraction"
 OR "heart failure with preserved ejection fraction"
 OR HFrEF
 OR HFmrEF
 OR HFpEF
 OR CHF
 OR ADHF
)
AND
(
 TITLE-ABS-KEY(
 Somalia
 OR Somaliland
 OR Puntland
 OR Mogadishu
 OR Hargeisa
 OR Garowe
 OR Garoowe
 OR Bosaso
 OR Bossaso
 OR Burao
 OR Burco
 OR Borama
 OR Boorama
 OR Baidoa
 OR Baydhabo
 OR Kismayo
 OR Kismaayo
 OR Galkayo
 OR Gaalkacyo
 OR Beledweyne
 OR Beletweyne
 OR Banadir
 OR Benadir
 OR Jubaland
 OR Galmudug
 OR Hirshabelle
 OR "Federal Republic of Somalia"
 )
 OR
 AFFIL(
 Somalia
 OR Somaliland
 OR Puntland
 OR Mogadishu
 OR Hargeisa
 OR Garowe
 OR Garoowe
 OR Bosaso
 OR Bossaso
 OR Burao
 OR Burco
 OR Borama
 OR Boorama
 OR Baidoa
 OR Baydhabo
 OR Kismayo
 OR Kismaayo
 OR Galkayo
 OR Gaalkacyo
 OR Beledweyne
 OR Beletweyne
 OR Banadir
 OR Benadir
 OR Jubaland
 OR Galmudug
 OR Hirshabelle
 )
)

# Search strategy 3: Embase

**Database/platform:** Embase (Elsevier) | **Date searched:** 27 July 2026 | **Records retrieved:** 51

(
 'heart failure'/exp
 OR 'heart failure':ti,ab,kw
 OR 'cardiac failure':ti,ab,kw
 OR 'myocardial failure':ti,ab,kw
 OR 'cardiac insufficiency':ti,ab,kw
 OR 'cardiac decompensation':ti,ab,kw
 OR 'congestive heart failure':ti,ab,kw
 OR 'congestive cardiac failure':ti,ab,kw
 OR 'acute heart failure':ti,ab,kw
 OR 'chronic heart failure':ti,ab,kw
 OR 'decompensated heart failure':ti,ab,kw
 OR 'acute decompensated heart failure':ti,ab,kw
 OR 'left ventricular failure':ti,ab,kw
 OR 'right ventricular failure':ti,ab,kw
 OR 'biventricular failure':ti,ab,kw
 OR 'systolic heart failure':ti,ab,kw
 OR 'diastolic heart failure':ti,ab,kw
 OR 'heart failure with reduced ejection fraction':ti,ab,kw
 OR 'heart failure with mildly reduced ejection fraction':ti,ab,kw
 OR 'heart failure with preserved ejection fraction':ti,ab,kw
 OR hfrEF:ti,ab,kw
 OR hfmrEF:ti,ab,kw
 OR hfpEF:ti,ab,kw
 OR chf:ti,ab,kw
 OR adhf:ti,ab,kw
)
AND
(
 'somalia'/exp
 OR somalia:ti,ab,kw
 OR somaliland:ti,ab,kw
 OR puntland:ti,ab,kw
 OR mogadishu:ti,ab,kw
 OR hargeisa:ti,ab,kw
 OR garowe:ti,ab,kw
 OR garoowe:ti,ab,kw
 OR bosaso:ti,ab,kw
 OR bossaso:ti,ab,kw
 OR burao:ti,ab,kw
 OR burco:ti,ab,kw
 OR borama:ti,ab,kw
 OR boorama:ti,ab,kw
 OR baidoa:ti,ab,kw
 OR baydhabo:ti,ab,kw
 OR kismayo:ti,ab,kw
 OR kismaayo:ti,ab,kw
 OR galkayo:ti,ab,kw
 OR gaalkacyo:ti,ab,kw
 OR beledweyne:ti,ab,kw
 OR beletweyne:ti,ab,kw
 OR banadir:ti,ab,kw
 OR benadir:ti,ab,kw
 OR jubaland:ti,ab,kw
 OR galmudug:ti,ab,kw
 OR hirshabelle:ti,ab,kw
 OR 'federal republic of somalia':ti,ab,kw
 OR somalia:ad
 OR somaliland:ad
 OR puntland:ad
 OR mogadishu:ad
 OR hargeisa:ad
 OR garowe:ad
 OR garoowe:ad
 OR bosaso:ad
 OR bossaso:ad
 OR burao:ad
 OR burco:ad
 OR borama:ad
 OR boorama:ad
 OR baidoa:ad
 OR baydhabo:ad
 OR kismayo:ad
 OR kismaayo:ad
 OR galkayo:ad
 OR gaalkacyo:ad
 OR beledweyne:ad
 OR beletweyne:ad
 OR banadir:ad
 OR benadir:ad
 OR jubaland:ad
 OR galmudug:ad
 OR hirshabelle:ad
)

# Backward citation searching

| **Date** | 3 August 2026 |
| --- | --- |
| **Sources searched** | Reference lists of all included studies |
| **Additional records identified** | One historical report |
| **Outcome** | The full text could not be retrieved; therefore, no additional study was included. |

# Search-yield summary

**Database records identified:** 111 (PubMed/MEDLINE, n = 27; Scopus, n = 33; Embase, n = 51). **Duplicates removed:** 56. **Records screened:** 55.

**Verification:** The uploaded export files contained 27 PubMed records, 33 Scopus records, and 51 Embase records, matching the numbers reported in the manuscript and PRISMA flow diagram.
