# Supplementary material for: Heart failure in Somalia: a scoping review of the epidemiology, etiology, clinical characteristics, management, outcomes, and evidence gaps
Source: Front Cardiovasc Med. 2026 Sep 28;13:1960696. doi: 10.3389/fcvm.2026.1960696 (PMC13656846; doi:10.3389/fcvm.2026.1960696)
Supplement: Supplementary file 2 [file Supplementaryfile2.docx]

**Additional file 2**

*Supplementary Tables 1–3*

**Heart Failure in Somalia: A Scoping Review**

**Supplementary Table 1. Methodological limitations, reporting inconsistencies, and possible cohort overlap among included studies**

| Study ID | Main methodological or reporting limitation | Possible overlap / generalisability concern |
| --- | --- | --- |
| S01 | Age was reported inconsistently; the 3.4% estimate is an emergency-unit proportion rather than population prevalence. | Single tertiary hospital in Mogadishu; recruitment period overlaps with other hospital-based studies. |
| S02 | Clinical HF diagnostic criteria were not fully described; EF categories overlap at 50%. | Single-centre selected record sample; possible overlap with other 2021 hospital cohorts. |
| S03 | Thirty-five HF cases were reported, but only 31 were assigned HFrEF/HFmrEF phenotypes; age differed between abstract and results. | AMI-selected cohort; findings are not representative of unselected HF. |
| S04 | HF diagnostic criteria and EF wording were inconsistent; sex count/percentage and age eligibility were internally inconsistent. | Single-centre record cohort with possible overlap with other Mogadishu HF studies. |
| S05 | HF findings came from a referred echocardiography subgroup; outcomes used different denominators. | Foreign military population; limited applicability to Somali civilians. |
| S06 | HF and acute pulmonary oedema were reported separately; blood-pressure definitions differed between methods and results. | Hypertensive-emergency-selected cohort; not generalisable to all HF. |
| S07 | HF count was review-derived from 27.1% of 59 CVD cases; risk-factor analyses applied to all CVD, not HF specifically. | Haemodialysis-selected cohort; not representative of the wider HF population. |
| S08 | Study flow could not be reconciled; repeat echocardiography was available for only 28 of 40 participants. | Selected HFrEF patients with LVT; not representative of all HFrEF. |
| S09 | Author-defined HFrEF included EF 41–49%; etiologies were not cross-tabulated for the HF subgroup. | Abnormal-echocardiogram registry; denominator is not all clinic attendees. |
| S10 | EF and SYNTAX data contained internal inconsistencies; treatment history was incomplete. | Angiography-selected referral cohort; CAD frequency cannot be generalised to all HF. |
| S11 | HALP cutoff was unclear; AUC was 0.511; several subgroup percentages were internally inconsistent. | Single-centre hospitalized acute-HF cohort; in-hospital outcomes only. |
| S12 | CALLY HR confidence interval crossed 1 despite p<0.05; model diagnostics contained placeholders and a simulated figure. | Single-centre hospitalized HF cohort; in-hospital follow-up only. |

**Supplementary Table 2. Heart-failure definitions, diagnostic approaches, phenotypes, aetiologies, and clinical findings reported in the included studies**

| Study ID | HF definition / ascertainment | Diagnostic methods | HF presentation / phenotype | Etiology or associated condition | Key clinical / diagnostic findings | Denominator and interpretation caution |
| --- | --- | --- | --- | --- | --- | --- |
| S01 | Framingham criteria; definite HF required >=2 major criteria or 1 major plus 2 minor criteria | Clinical assessment and echocardiography; BNP not used | HFpEF 74/155 (47.7%); HFrEF 54/155 (34.9%); HFmrEF 27/155 (17.4%) | HHD 70/155 (45.2%); IHD 40/155 (25.8%); VHD 20/155 (12.9%); cardiomyopathy 12/155 (7.7%); arrhythmia 9/155 (5.8%); CHD 4/155 (2.6%) | Hypertension history 102/155 (65.8%); median EF 45% (range 30-50) | The reported 3.4% is a hospital emergency-unit proportion (155/4,500), not national or population prevalence; age is inconsistently reported. |
| S02 | HF identified from hospital records/ICD-10; HFrEF EF <40%; mid-range EF 40-50%; HFpEF EF >50% | Echocardiographic EF categories and laboratory assessment of anemia/iron/folate/B12 | HFpEF 95/200 (47.5%); HFrEF 68/200 (34.0%); mid-range EF 37/200 (18.5%) | Hypertension 99/200 (49.5%); IHD 56/200 (28.0%); VHD 34/200 (17.0%); cardiomyopathy 11/200 (5.5%) | Anemia 59/200 (29.5%); iron-deficiency anemia 37/200 (18.5%) | The EF threshold wording overlaps at 50%; the study does not provide detailed clinical HF diagnostic criteria. |
| S03 | HF was reported as an AMI complication; EF phenotypes were determined by echocardiography | AMI diagnosis by clinical presentation, ECG, biomarkers/ESC guidance; all 97 underwent echocardiography | HF occurred in 35/97 patients (36.1%); among these 35 HF cases, 19 had HFrEF and 12 had HFmrEF, while the phenotype of four cases was not reported. | Acute MI/ischemic injury was the parent clinical condition | HF occurred in 27/73 STEMI (37.0%) and 8/24 NSTEMI (33.3%) | The 35 total HF cases exceed the 31 categorized as HFrEF/HFmrEF; do not infer the phenotype of the remaining 4. Other AMI risk factors are not HF-specific. |
| S04 | Previously diagnosed HF from electronic records; paper classifies patients as HFrEF or HFpEF | Echocardiographic EF categories plus TSH, FT3, and FT4 assays | HFrEF 147/250 (58.8%); HFpEF 103/250 (41.2%) | Among 89 with thyroid dysfunction: hypertension 40.4%, IHD 31.5%, VHD 20.2%, cardiomyopathy 9.0% | Thyroid dysfunction 89/250 (35.6%); in the thyroid subgroup HFrEF 64/89 (71.9%) and HFpEF 25/89 (28.1%) | Methods contain inconsistent EF wording for HFpEF and inconsistent age eligibility ('regardless of age' but <30 excluded). Sex count/percentage also do not match exactly. |
| S05 | Outbreak case definition: acute lower-limb edema plus >=1 symptom; right-sided HF identified by echocardiographic changes in the hospitalized sub-study | Clinical assessment, echocardiography, cardiac catheterization in 10, extensive laboratory testing, thiamine testing | Right-sided HF changes 6/31 (19.4%); pulmonary hypertension 4/31 | Thiamine-deficiency wet beriberi caused by restricted diet | All 33 hospitalized patients had edema; 22/33 palpitations; 14/33 dyspnea; 6 required higher-level cardiorespiratory care | The population was foreign AU troops stationed in Mogadishu, not Somali civilians. HF data derive from the referred hospitalized/echo subgroup, not all 241 outbreak cases. |
| S06 | Hypertensive emergency included congestive HF or acute pulmonary edema as acute target-organ damage; diagnoses were clinical and test-supported | Blood chemistry, ECG, echocardiography, CT/MRI, ultrasound as appropriate | Acute HF 32/70 hypertensive emergencies (45.7%); acute pulmonary edema 23/70 (reported separately) | Severe hypertension/hypertensive emergency | Hypertensive emergencies 70/128; urgencies 58/128 | Do not combine acute HF and pulmonary edema automatically. Methods use >180/120 mmHg, but results describe hypertensive emergency using >140/90 plus target-organ damage. |
| S07 | HF defined as abnormal LV filling/mitral E:A pattern and/or systolic dysfunction with EF <50% | Hospital records, ECG, two-dimensional and Doppler echocardiography | Approximately 16 HF cases among 59 patients with at least one CVD | ESRD and routine hemodialysis | CVD occurred in 59/200 (29.5%); HF represented 27.1% of CVD diagnoses | The 27.1% denominator is the 59 CVD cases, not all 200. The paper does not state the HF count directly; approximately 16 is derived from 27.1% of 59. Risk-factor analyses concern all CVD, not HF specifically. |
| S08 | Eligibility required echocardiographically confirmed LVT, clinical HF, and LVEF <40% | Two-dimensional transthoracic echocardiography; Simpson biplane EF | HFrEF with LVT; mean LVEF 25.25 ± 6.97%; LVEF <=25% in 24/40 (60%) | Ischemic cardiomyopathy 23/40 (57.5%); non-ischemic cardiomyopathy 17/40 (42.5%) | LVT location: apex 21/40, inferior wall 11/40, anterior wall 8/40; LV aneurysm 15/40 | The study includes only HFrEF patients with LVT, not all HFrEF. The flowchart cannot be reconciled: 810 total, 570 excluded, 240 without LVT, and 40 included sum to 850. |
| S09 | Author-defined HFrEF required HF risk factors/abnormal ECG/signs or symptoms plus EF <50%; EF 41-49% was called mildly reduced LV systolic dysfunction | Echocardiography performed by experienced licensed echocardiographers | Author-defined HFrEF 351/1,140 (30.8%); 59/1,140 had EF 41-49% and 292/1,140 had EF <=40% | HHD, IHD, VHD, DCM and other CVDs were reported for the full abnormal-echo cohort | Median EF in the full cohort 60% (IQR 40-65) | 30.8% is among 1,140 abnormal echocardiograms, not all 6,782 attendees. The authors' HFrEF category includes conventional HFmrEF. Etiologies were not cross-tabulated specifically within the HF subgroup. |
| S10 | Confirmed HFrEF (LVEF <40%) or HFmrEF (41-49%) in patients undergoing coronary angiography for suspected CAD | Biplane Simpson echocardiography and invasive coronary angiography; SYNTAX scoring | Systolic HF/HFmrEF; mean LVEF 28.6 ± 6% | Angiographically detected CAD 90/169 (53.3%); severe CAD 39/169 (23.1%) | Normal arteries 79/169; minor CAD 35/169; moderate CAD 16/169; SVD 51, DVD 24, TVD 13, left-main involvement 2 | This is an angiography-selected referral cohort; 53.3% is not the prevalence of CAD in all Somali HF. EF values are reversed between narrative and Table 1, and SYNTAX reporting has denominator inconsistencies. |
| S11 | Adults hospitalized with acute HF confirmed according to ESC guidelines and with complete HALP laboratory data | Clinical record review; routine laboratory values; proBNP reported as elevated in 216/222 | Acute hospitalized HF; EF phenotype not reported | Nutritional/inflammatory risk measured by HALP; hypertension 118/222, diabetes 123/222, CKD 29/222 | HALP >= study cutoff in 23/222; age >=65 in 42/222 | The actual HALP cutoff is not reported, and the paper alternately calls it previously validated and internally ROC-derived. No etiologic or EF-phenotype data are presented. |
| S12 | ESC-based diagnosis requiring compatible symptoms/signs, elevated proBNP, and echocardiographic dysfunction | proBNP, biplane Simpson echocardiography, CRP, albumin, lymphocyte count | HFrEF 38.2% (~84); HFmrEF 25.5% (~56); HFpEF 36.4% (~80) | Inflammatory/nutritional risk assessed with CALLY; diabetes and hypertension common across phenotypes | Non-survivors had lower CALLY (1.27 ± 0.72 vs 1.71 ± 0.60) and higher CRP | Phenotype counts are inferred from percentages because exact counts are in unavailable supplementary material. Methods retain a [Hospital Name] placeholder. |

**Supplementary Table 3. Management, clinical outcomes, follow-up, and prognostic indicators reported in the included studies**

| Study IERRHGGHGID | Medical treatment | Procedures / diagnostic interventions | Length of stay | Mortality | Other outcomes | Follow-up | Interpretation caution |
| --- | --- | --- | --- | --- | --- | --- | --- |
| S01 | Not reported | Echocardiography used for classification | Not reported | Not reported | Not reported | Not reported | This study focused on baseline characteristics and etiology rather than treatment or outcomes. |
| S02 | Not reported | Laboratory characterization of anemia and nutrient deficiencies | Not reported | Not reported | Anemia 59/200; iron deficiency was the predominant anemia pattern | Not reported | No HF treatment or clinical outcome data were reported. |
| S03 | AMI medications were reported for the full AMI cohort, not separately for HF cases; therefore not used as HF management evidence | Echocardiography in all AMI patients; reperfusion/AMI care reported for parent cohort | Not reported for HF subgroup | Not reported for HF subgroup | HF 35/97; arrhythmia and cardiogenic shock were parent-cohort complications | Not reported | Do not attribute aspirin, clopidogrel, LMWH, thrombolysis, or other AMI treatments specifically to the HF subgroup. |
| S04 | Among the 89 HF patients with thyroid dysfunction: ACE inhibitor 97.8%, beta-blocker 98.9%, spironolactone 77.5%, furosemide/Lasix 80.7%; thyroid drugs 19.1%, levothyroxine 38.2% | Thyroid-function testing and echocardiographic EF classification | Not reported | Not reported | Not reported | Not reported | Medication percentages apply to the thyroid-dysfunction subgroup, not all 250 HF patients. |
| S05 | Intramuscular thiamine for symptomatic cases; mass supplementation initially 10 mg daily for 2 weeks then 1 mg daily for 4 weeks; dietary diversification | Echocardiography in 31; cardiac catheterization in 10; extensive infectious/toxic/nutritional testing | Not consistently reported | 4/241 outbreak cases (1.7%); 4/33 in the Nairobi hospitalized subgroup | 29/33 hospitalized cases recovered and were discharged; 6 required higher-level cardiorespiratory care; no deaths after mass supplementation began | Outbreak observed through 1 May 2010 | Outcomes are reported across different denominators (241 outbreak cases, 33 hospitalized, 31 echo patients). |
| S06 | Antihypertensive treatment was reported for the full hypertensive-crisis cohort, not specifically for acute-HF cases | Clinical and diagnostic evaluation for target-organ damage | Not reported for HF subgroup | Not reported for HF subgroup | Acute HF 32/70 hypertensive emergencies; acute pulmonary edema 23/70 | Not reported | Do not present full-cohort furosemide/nitroglycerin/captopril use as HF-specific treatment. |
| S07 | Not reported for HF subgroup | Hemodialysis plus ECG/echo assessment | Not reported | Not reported | HF was the most common CVD diagnosis among the 59 CVD cases | Not reported | The paper reports predictors for any CVD, not HF-specific management or outcomes. |
| S08 | Long-term anticoagulation in 37/40: warfarin 19/40, rivaroxaban 8/40, dabigatran 10/40; two died before oral anticoagulation and one had bleeding diathesis | Repeat echocardiography in 28/40 | Not reported | 12/40 (30%) within the first year | Stroke 8/40 (20%): 6 ischemic, 2 hemorrhagic; PAD 6/40 (15%); complete LVT resolution 17/28 (60.7% of those reassessed), persistence 11/28 (39.3%) | First year after LVT diagnosis | The paper reports resolution percentages using the full n=40 denominator; use 28 as the repeat-echo denominator. Four of 11 persistent thrombi decreased in size. |
| S09 | Not reported | Echocardiographic registry | Not reported | Not reported | Not reported | Not reported | No treatment, readmission, surgery, or mortality outcomes were reported. |
| S10 | Treatment history was not consistently documented | Coronary angiography in all 169; SYNTAX scoring | Not reported | Not reported | Angiographic CAD burden and vessel distribution | Not reported | This study provides diagnostic/anatomic data rather than management or outcomes. |
| S11 | Not reported | HALP calculation, routine laboratory testing, ROC and regression modeling | Long stay >7 days in 86/222 (38.7%) | 21/222 (9.5%) in hospital | HALP >= cutoff: adjusted OR 10.19 (95% CI 2.49-41.63) for prolonged stay; prior stroke OR 8.44 (1.15-61.88); HALP AUC 0.511 | In-hospital only | Published subgroup mortality percentages are mathematically wrong: 4/136 = 2.9% and 17/86 = 19.8%, not 2.1% and 65.4%. Many long-stay percentages appear to use n=84 rather than 86. |
| S12 | Specific treatment frequencies not reported | CALLY calculation, echocardiography, proBNP, Cox modeling and Kaplan-Meier analysis | Survivors 3.05 ± 1.06 days; non-survivors 4.96 ± 1.84 days; phenotype medians: HFpEF 5 days, HFrEF 4, HFmrEF 4 | 26/220 (11.8%) in hospital; crude phenotype mortality: HFpEF 15.0%, HFmrEF 10.7%, HFrEF 9.5% | Age The source article reported a CALLY estimate of HR 0.495 (95% CI 0.281–1.010; p = 0.047). Because the confidence interval included 1.0 despite the reported p value being below 0.05, the statistical results were internally inconsistent and the association was considered inconclusive. | In-hospital; 30-day readmission was planned but not reported | The CALLY CI crosses 1.0. Model diagnostics contain [insert] placeholders; Figure 3 is labelled simulated; the hospital-stay Cox coefficient is unstable. |
