# Supplementary material for: Heart failure in Somalia: a scoping review of the epidemiology, etiology, clinical characteristics, management, outcomes, and evidence gaps
Source: Front Cardiovasc Med. 2026 Sep 28;13:1960696. doi: 10.3389/fcvm.2026.1960696 (PMC13656846; doi:10.3389/fcvm.2026.1960696)
Supplement: Supplementary file 3 [file Supplementaryfile3.docx]

**Additional file 3**

*PRISMA-ScR checklist – working completion sheet*

**Note:** Manuscript section locations are mapped below. Add final page numbers after the manuscript layout is fixed.

| Item | Section/topic | Checklist requirement (abridged) | Location in manuscript | Page |
| --- | --- | --- | --- | --- |
| 1 | Title | Identify the report as a scoping review. | Title |  |
| 2 | Structured summary | Provide a structured summary including background, objectives, eligibility criteria, sources, charting, results, and conclusions. | Abstract |  |
| 3 | Rationale | Describe the rationale in the context of what is already known. | Introduction, paragraphs 1–4 |  |
| 4 | Objectives | State the review questions and objectives using PCC or equivalent. | Introduction final paragraph; Methods: Review question and eligibility criteria |  |
| 5 | Protocol and registration | Indicate whether a protocol exists and provide registration information. | Methods: Protocol and registration |  |
| 6 | Eligibility criteria | Specify characteristics used to determine eligibility and rationale. | Methods: Review question and eligibility criteria |  |
| 7 | Information sources | Describe all information sources and the last search date. | Methods: Information sources |  |
| 8 | Search | Present a full electronic search strategy for at least one database. | Additional file 1 – exact strings still required |  |
| 9 | Selection of sources | State the process for selecting sources of evidence. | Methods: Selection of sources of evidence |  |
| 10 | Data charting process | Describe methods of charting data and verification. | Methods: Data charting process |  |
| 11 | Data items | List and define all variables sought. | Methods: Data items |  |
| 12 | Critical appraisal | Describe appraisal methods and how appraisal was used, when conducted. | Methods: Critical appraisal of sources of evidence |  |
| 13 | Synthesis of results | Describe methods of handling and summarising charted data. | Methods: Synthesis and presentation of results |  |
| 14 | Selection of sources of evidence | Report numbers screened, assessed, and included, ideally with a flow diagram. | Results: Study selection; Figure 1 |  |
| 15 | Characteristics of sources | Present characteristics of each source and citations. | Results: Characteristics of included studies; Table 1 |  |
| 16 | Critical appraisal within sources | Present appraisal results, when conducted. | Not formally conducted; methodological characteristics charted descriptively |  |
| 17 | Results of individual sources | Present relevant charted data for each source. | Table 1; Supplementary Tables 1–3 |  |
| 18 | Synthesis of results | Summarise and/or present charting results as related to objectives. | Results narrative; Table 1; Supplementary Tables 1–3 |  |
| 19 | Summary of evidence | Summarise main results, concepts, and relevance. | Discussion: Principal findings |  |
| 20 | Limitations | Discuss limitations of the scoping-review process. | Discussion: Strengths and Limitations |  |
| 21 | Conclusions | Provide a general interpretation and implications/future steps. | Conclusions; Discussion: Health-System Implications and Research Priorities |  |
| 22 | Funding | Describe funding for the included evidence and for the review. | Declarations: Funding; funding of individual sources not systematically reported |  |
